# Comparison of colorectal cancer screening between people with and without disability: a nationwide matched cohort study

**DOI:** 10.1186/s12889-021-11105-z

**Published:** 2021-06-02

**Authors:** Chun-Ming Liao, Wen-Hao Huang, Pei-Tseng Kung, Li-Ting Chiu, Wen-Chen Tsai

**Affiliations:** 1 Department of Public Health, China Medical University, Taichung, Taiwan; 2 Genetic and Rare Disease Center, China Medical University Hospital, Taichung, Taiwan; 3 Department of Gastroenterology and Hepatology, Yee Zen General Hospital, Taoyuan City, Taiwan; 4 Department of Healthcare Administration, Asia University, Taichung, Taiwan; 5 Department of Medical Research, China Medical University Hospital, China Medical University, Taichung, Taiwan; 6 Department of Health Services Administration, China Medical University, No. 100, Sec. 1, Jingmao Road, Beitun District, Taichung, 406040 Taiwan

**Keywords:** Disability, Colorectal cancer, Colorectal cancer screening

## Abstract

**Background:**

The World Health Organization has recognized that people with disability are among the most marginalized in the world. This study’s objective was to investigate the differences in the probability of colorectal cancer (CRC) screening with faecal immunochemical testing (FIT) between people with disability and without disability in Taiwan.

**Methods:**

The study participants included people with and without disability from the Disability Registration Database (2012) and the National Health Insurance Research Database (2009–2012). The study included 50- to 69-year-olds with and without disability who were screened from 2011 to 2012 and were alive in 2012. There were 16 categories of disability. After propensity score matching (PSM) between the two groups, conditional logistic regression analysis with control variables was used to investigate the odds ratio (OR) that people with or without disability would undergo CRC screening.

**Results:**

The percentage of people with disability receiving CRC screening was 21.84%, and the highest rate of those receiving CRC screening (38.72%) was found in people with intractable epilepsy, whose OR was 1.47 times that of people with moving functional limitation (95% confidence interval (CI) = 1.17–1.85). The results showed that the probability of CRC screening in people with disability was lower than that in people without disability (OR = 0.88, 95%CI = 0.87–0.89). The probability of receiving CRC screening differed between people with different categories of disability.

**Conclusions:**

Although the probability of CRC screening in the four categories of disability was higher than that in the general population, overall, people with disability were less likely than people without disability to undergo CRC screening. Health inequalities still exist under National Health Insurance in Taiwan.

## Background

A World Health Organization (WHO) survey in 2010 showed that the number of people with disability exceeds one billion globally (approximately 15.6–19.4% of the global population) and has been increasing in recent years [1]. The WHO report also noted that people with disability are one of the most marginalized groups in the world, and they generally suffer from poor health, difficulty accessing education, and low socioeconomic status, though these inequities often can be overcome through active effort [2]. The WHO has found that people with disability are twice as likely as the general population (without disability) to be denied access to health care and up to three times as likely to suffer from inappropriate medical treatment [2]. In Taiwan, the number of people with disability reached 1.17 million in 2014 (approximately 5% of the population), and due to the ageing of the population, this number has been increasing significantly over the years [3].

In the past decade, cancer has been the leading cause of death in Taiwan, and colorectal cancer (CRC) has the highest incidence among all cancers. Therefore, since 2004, Taiwan’s Ministry of Health and Welfare has been promoting free faecal immunochemical testing (FIT) for CRC and other cancer screenings for people aged 50 to 69 years (after June 1, 2013, the age range was changed to 50–74 years), and the screening rate within 2 years has risen to approximately 40% in recent years [3]. A meta-analysis showed that people with disability are not significantly less likely to undergo FIT than people without disability [4]. Another study using public data from the National Health Interview Survey also showed that people with disability were less likely to undergo CRC screening in 1998, but there was no difference in 2010 [5]. In contrast to the above viewpoints, one systematic review in 2020 screened more than 5000 articles and, after analysing 18 of them, concluded that although people with disability share many of the same barriers as people without disability, their utilization rate of cancer treatment is not higher than that of people without disability but their utilization rate of cancer screening is much lower [6]. In Taiwan, where free health checks have been available to adults for more than a decade, several studies have shown that people with disability are significantly less likely to receive free health checks than others [7–9]. The probability of cervical cancer screening in people with disability is lower than that in people without disability [10–12]. The probability of breast cancer screening in people with disability is also lower than that in people without disability, especially for women with mental disorders [13]. Few international articles have compared CRC screening between people with and without disability, and the conclusions are not consistent. In Taiwan, there are no articles on CRC screening among people with disability.

A large US study (> 4 million subjects) showed that the probability of CRC screening (83.1%) in the 51–75-year-old age group reached the target of ≥80%, contributing to a significant reduction in the mortality risk of CRC [14, 15]. In fact, the probability of CRC screening varies widely among different ethnic groups, and inconsistent screening rates have caused difficulties in the implementation of public health policies [16]. Although the screening rate (once every two years) for CRC in Taiwan is approximately 40% [3], a time series analysis taking Taiwanese people as the sample showed that over the years, due to the implementation of National Health Insurance (NHI) and medical technology advances in Taiwan, a significant reduction in the mortality rate of CRC has been achieved [17]. Other studies using different statistical methods to determine the CRC screening rate have also drawn the same conclusion; that is, the death risk of CRC decreases year by year [18, 19]. Furthermore, increasing the screening rate allows early treatment in patients and a better survival rate, a conclusion drawn by many cancer-related studies [20–22].

The objective of this study was to investigate the differences in the probability of CRC screening between people with and without disability in Taiwan.

## Methods

### Data sources

In this study, the Disability Registration Database (2012) of the Ministry of the Interior of Taiwan and the National Health Insurance Research Database (NHIRD) (2009–2012) provided by the Ministry of Health and Welfare of Taiwan were used as the population source and were combined with national catastrophic illness/injury data as of December 2012. The total number of people under Taiwan’s NHI was 23.621 million [23], and the coverage rate was 99.9% [24]. The data from this study are representative of the Taiwanese population.

The Disability Registration Database in Taiwan is established and maintained by the Ministry of the Interior of Taiwan. Under Taiwan’s Physically and Mentally Disabled Citizens Protection Act, after assessment by a government-certified professional physician, people with disability must apply to the Department of Social Welfare of a county or city, obtain a permit, and notify the Ministry of the Interior of Taiwan to complete the registration.

Patient identifications in the NHIRD and the Disability Registration Database were scrambled to ensure privacy. All patients without identifications for public use and personal privacy were protected in this study. The study guidelines followed for this study were in accordance with the Declaration of Helsinki. The Research Ethics Committee of Dali JEN-AI Hospital waived the informed consent in this study and approved this study (IRB No: 105–14).

### Participants

According to Taiwan’s *Physically and Mentally Disabled Citizens Protection Act*, this study classified 16 categories of disability (moving functional limitation, internal organ function loss and related disabilities, hearing impairment, multiple disabilities, chronic mental health conditions, visual impairment, intellectual and developmental disability, dementia, vocal and speech impairment, motion and balance impairment, facial disfigurements, intractable epilepsy, rare diseases, autism, persistent vegetative state, and other (chromosomal abnormalities, inborn errors of metabolism, and birth defects)) and four severity levels of disability (mild, moderate, severe, and profound). People in a persistent vegetative state (PVS) are not suitable for the purpose of this study, so this category was excluded.

**Fig. 1:**
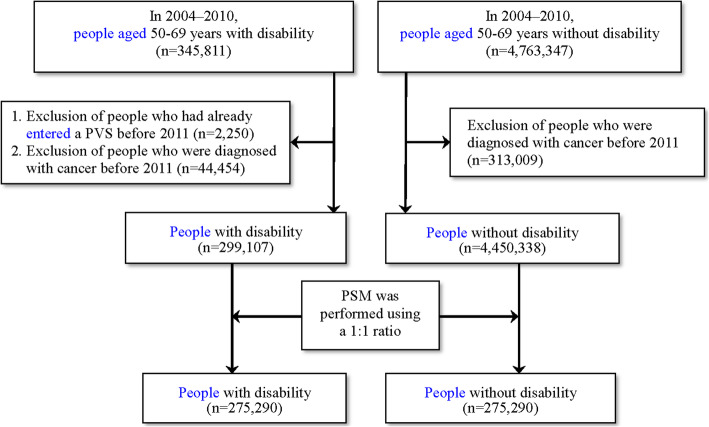
Process of selecting the study participants meeting the CRC screening criteria

The objective of this study was to calculate the differences in the probability of CRC screening (FIT) between people with and without disability. Among the people aged 50–69 years who received a free screening under NHI between 2011 and 2012, there were 345,811 people with disability and 4,763,347 people without disability. Among those with disability, 2250 people who had already entered a PVS and 44,454 people who were diagnosed with cancer before 2011 were excluded. Among the people without disability, 313,009 people who had cancer before 2011 were excluded. Finally, 299,107 people with disability and 4,450,338 people without disability were included in this study (Fig. 1).

### Variable descriptions and definitions

Because free CRC screening with FIT in Taiwan is conducted once every 2 years for eligible people [3], the dependent variable was whether the study participants received CRC screening in 2011 or 2012. The independent variables were category of disability (15 types), severity of disability (four levels), and whether one did or did not have a disability (i.e., covering those with or without disability who met the CRC screening criteria), and the control variables were sex (male or female), age (50–59 or 60–69 years), and socioeconomic status (monthly salary, including low income, ≤ 17,280 NT$, 17,281–22,800 NT$, 22,801–28,800 NT$, 28,801–36,300 NT$, and ≥ 36,301 NT$). The environmental factor was represented by the urbanization level of the residential area. Based on a 2006 study by Liu et al. [25], the 359 townships in Taiwan were classified into seven levels. Townships with the highest urbanization level were classified as the first level, and those with the lowest urbanization level were classified as the seventh level [25]. Health condition was measured by the comorbidity severity of each subject. According to the Charlson comorbidity index (CCI) modified by Deyo et al. [26], the ICD-9-CM primary and secondary codes of each subject were converted into numerical weight scores to represent the comorbidity severity, which included 0, 1, 2, and ≥ 3 points in this study; the higher the score, the more severe the comorbidity is. The preventive health care behaviour of the study participants was represented by their participation in free dental cleaning and the use of free adult health examinations under NHI. Taiwan National Health Insurance offers free preventive dental calculus cleaning once every 6 months for all people. Thus, the receipt of dental calculus cleaning in 2011–2012 was used as a variable. Taiwan’s NHI also provides a free adult health examination once every 3 years for people aged between 40 and 65 years and once every year for people aged 65 years and above. The receipt of a free adult health examination under NHI in 2011–2012 was used as the second variable.

### Analytical methods

This study was a retrospective cohort study. SAS 9.4 (SAS Institute Inc., Cary, NC) was used for statistical analysis.

First, the probability of receiving CRC screening for people with different categories of disability and different severity levels of disability was analysed, and the chi-squared (χ^2^) test was used to compare the probability of receiving CRC between two groups to determine whether there were significant differences in the distribution of people (*P* <  0.05). Then, the probability of receiving CRC screening was analysed by conditional logistic regression after adding the control variables (seven variables) to investigate the odds ratio (OR) and factors relevant to CRC screening in people with different categories of disability and different severity levels of disability.

To calculate the differences in the probability of CRC screening between people with and without disability who met the screening criteria, since people with disability (observation group) and people without disability (control group) were not randomly enrolled, propensity score matching (PSM) was used to reduce the selection bias between the two groups [27]. First, we built a multivariate logistic regression model with relevant factors, including demographic characteristics, socioeconomic status, environmental factors, and health conditions, to calculate the predicted probability for people with disability. We then used the closest propensity score to match the people with and without disability at a ratio of 1:1 to generate the observation and control groups. If a person with disability (observation group) could not be matched with any person without disability (control group) via the greedy algorithm, then this person was excluded from the observation group. A chi-squared (χ^2^) test was performed to test the people with disability (observation group) and people without disability (control group) to confirm that the distribution of people for each variable was not significantly different between the two groups (*P* > 0.05). Furthermore, before comparing the likelihood of undergoing CRC screening between people with and without disability, the observation and control groups were tested by the χ^2^ test to determine whether there was a significant difference in the distribution of each variable between the two groups (*P* <  0.05). Next, in Model A with the seven control variables, conditional logistic regression was used to analyse the probability of CRC screening in people with and without disability to investigate the OR and its influencing factors. Finally, in Model B, the people with disability were divided into 15 categories. After adding the control variables (seven variables), the OR and relevant factors of receiving CRC screening in the general population and people in the 15 categories of disability were investigated separately.

## Results

Table 1 shows the comparison of the number of people with disability who did and did not undergo CRC screening, and the χ^2^ test was used to compare the two groups. The results showed that the number of people with disability who underwent CRC screening (65,329, 21.84%) was lower than that of people with disability who did not receive CRC screening (233,778, 78.16%). Among the categories of disability, the percentage of people receiving CRC screening was highest among people with intractable epilepsy (139 patients, 38.72%) and lowest among people with dementia (450 patients, 11.86%). With the increase in severity level, the percentage of people receiving CRC screening tended to decrease, from 27.05% (mild) to 14.13% (profound). The percentage of people receiving CRC screening decreased as comorbidity severity increased, from 23.61% (CCI = 0) to 14.94% (CCI ≥ 3).

**Table 1:** The Use of FIT for CRC Screening in People with Disability

				No FIT		FIT^a^		
		N	%	N	%	N	%	*p*-value
**Total**		299,107	100.00	233,778	78.16	65,329	21.84	
**Category of disability**								< 0.001
Moving functional limitation		144,953	48.46	111,976	77.25	32,977	22.75	
Internal organ function loss and related disabilities		41,344	13.82	33,518	81.07	7826	18.93	
Hearing impairment		27,422	9.17	20,296	74.01	7126	25.99	
Multiple disabilities		25,472	8.52	21,753	85.4	3719	14.60	
Chronic mental health conditions		24,642	8.24	17,517	71.09	7125	28.91	
Visual impairment		17,569	5.87	13,985	79.6	3584	20.40	
Intellectual and developmental disability		9671	3.23	8195	84.74	1476	15.26	
Dementia		3794	1.27	3344	88.14	450	11.86	
Vocal and speech impairment		2455	0.82	1914	77.96	541	22.04	
Motion and balance impairment		803	0.27	621	77.33	182	22.67	
Facial disfigurements		437	0.15	299	68.42	138	31.58	
Intractable epilepsy		359	0.12	220	61.28	139	38.72	
Rare diseases		85	0.03	66	77.65	19	22.35	
Autism		65	0.02	50	76.92	15	23.08	
Other^b^		36	0.01	24	66.67	12	33.33	
**Severity of disability**								< 0.001
Mild		109,180	36.50	79,646	72.95	29,534	27.05	
Moderate		97,543	32.61	75,641	77.55	21,902	22.45	
Severe		50,611	16.92	42,619	84.21	7992	15.79	
Profound		41,773	13.97	35,872	85.87	5901	14.13	
**Sex**								< 0.001
Male		173,959	58.16	138,078	79.37	35,881	20.63	
Female		125,148	41.84	95,700	76.47	29,448	23.53	
**Age (years)**								< 0.001
50–59		175,332	58.62	123,047	70.18	52,285	29.82	
60-69		123,775	41.38	110,731	89.46	13,044	10.54	
**Monthly salary (NT$**^**c**^**)**								< 0.001
low income	16,331		5.46	13,098	80.20	3233	19.80	
≤ 17,280	17,382		5.81	14,585	83.91	2797	16.09	
17,281-22,800	124,769		41.71	100,029	80.17	24,740	19.83	
22,801-28,800	56,207		18.79	44,187	78.61	12,020	21.39	
28,801-36,300	40,823		13.65	30,782	75.40	10,041	24.60	
≥ 36,301	43,595		14.58	31,097	71.33	12,498	28.67	
**Urbanization level of residence area**								< 0.001
Level 1	61,666		20.62	48,453	78.57	13,213	21.43	
Level 2	86,553		28.94	65,913	76.15	20,640	23.85	
Level 3	46,154		15.43	36,338	78.73	9816	21.27	
Level 4	55,312		18.49	43,918	79.40	11,394	20.60	
Level 5	10,177		3.40	7951	78.13	2226	21.87	
Level 6	21,069		7.04	16,581	78.70	4488	21.30	
Level 7	18,176		6.08	14,624	80.46	3552	19.54	
**CCI**^**d**^								< 0.001
0	131,068		43.82	100,118	76.39	30,950	23.61	
1	63,076		21.09	47,143	74.74	15,933	25.26	
2	42,825		14.32	33,660	78.60	9165	21.40	
≥ 3	62,138		20.77	52,857	85.06	9281	14.94	
**Dental calculus cleaning**								< 0.001
No	227,891		76.19	187,709	82.37	40,182	17.63	
Yes	71,216		23.81	46,069	64.69	25,147	35.31	
**Adult health examination**								< 0.001
No	221,087		73.92	184,436	83.42	36,651	16.58	
Yes	78,020		26.08	49,342	63.24	28,678	36.76	

a. *FIT* faecal immunochemical testing; b. Other: chromosomal abnormalities, inborn errors of metabolism, congenital defects; c. *NT$* New Taiwan dollar; d. *CCI* Charlson comorbidity index.

Table 2 shows the results of the logistic regression analysis. With the control variables considered, compared to the OR in people with moving functional limitation, the ORs of receiving CRC screening in people with intractable epilepsy (OR = 1.47 (i.e., 47% higher), 95% confidence interval (CI) = 1.17–1.85), internal organ function loss and related disabilities (OR = 1.34, 95%CI = 1.29–1.39), chronic mental health conditions (OR = 1.29, 95%CI =1.24–1.33), and hearing impairment (OR = 1.22, 95%CI = 1.18–1.26) were higher, while those of people with intellectual and developmental disability (OR = 0.72, 95%CI = 0.68–0.77) and dementia (OR = 0.79, 95%CI = 0.71–0.88) were lower. Compared to people with mild disability, the OR of receiving CRC screening decreased with the increase in the disability severity. The OR of receiving CRC screening in people with disability also decreased with the increase in the severity of comorbidities.

**Table 2:** Factors Associated with the Use of FIT for CRC Screening in People with Disability

	OR	95% CI		*p-*value
**Category of disability**				
Moving functional limitation (ref)	1.00	–	–	–
Internal organ function loss and related disabilities	1.34	1.29	1.39	< 0.001
Hearing impairment	1.22	1.18	1.26	< 0.001
Multiple disabilities	1.00	0.96	1.05	0.885
Chronic mental health conditions	1.29	1.24	1.33	< 0.001
Visual impairment	1.03	0.99	1.07	0.197
Intellectual and developmental disability	0.72	0.68	0.77	< 0.001
Dementia	0.79	0.71	0.88	< 0.001
Vocal and speech impairment	1.08	0.97	1.20	0.152
Motion and balance impairment	1.14	0.95	1.36	0.163
Facial disfigurements	1.17	0.94	1.45	0.166
Intractable epilepsy	1.47	1.17	1.85	0.001
Rare diseases	0.92	0.53	1.61	0.771
Autism	0.89	0.48	1.65	0.716
Other^a^	1.77	0.85	3.69	0.129
**Sex**				
Male (ref)	1.00	–	–	–
Female	1.19	1.16	1.21	< 0.001
**Age (years)**				
50–59 (ref)	1.00	–	–	–
60–69	0.23	0.22	0.23	< 0.001
**Monthly salary (NT$**^**b**^**)**				
Low income (ref)	1.00	–	–	–
≤ 17,280	0.89	0.83	0.94	< 0.001
17,281-22,800	1.07	1.02	1.12	0.007
22,801-28,800	1.05	1.00	1.10	0.048
28,801-36,300	1.17	1.11	1.23	< 0.001
≥ 36,301	1.40	1.33	1.47	< 0.001
**Urbanization level of residence area**				
1 (ref)	1.00	–	–	–
2	1.14	1.11	1.17	< 0.001
3	1.04	1.01	1.08	0.010
4	1.07	1.03	1.10	< 0.001
5	1.34	1.26	1.42	< 0.001
6	1.21	1.16	1.27	< 0.001
7	1.13	1.08	1.18	< 0.001
**CCI**^**c**^				
0 (ref)	1.00	–	–	–
1	1.24	1.21	1.27	< 0.001
2	1.22	1.19	1.26	< 0.001
≥ 3	1.04	1.00	1.07	0.027
**Severity of disability**				
Mild (ref)	1.00	–	–	–
Moderate	0.85	0.84	0.87	< 0.001
Severe	0.62	0.60	0.64	< 0.001
Profound	0.56	0.54	0.59	< 0.001
**Dental calculus cleaning**				
No (ref)	1.00	–	–	–
Yes	1.88	1.84	1.92	< 0.001
**Adult health examination**				
No (ref)	1.00	–	–	–
Yes	3.32	3.25	3.39	< 0.001

a. Others: chromosomal abnormalities, inborn errors of metabolism, and congenital defects; b. *NT$* New Taiwan dollar; c. *CCI* Charlson comorbidity index.

To understand the differences in the probability of CRC screening between people with and without disability, based on a rigorous study design, this study performed 1:1 PSM between people with and without disability. After matching, there were 275,290 participants in each group (Fig. 1). Testing showed no significant difference in the distribution of each variable between the two groups (*P* > 0.05) (Table 3).

**Table 3:** Distribution after Matching between People with and without Disability

			Without disability		With disability		
	N	%	N	%	N	%	*p*-value
**Total**	550,580	100.00	275,290	50.00	275,290	50.00	
**Sex**							0.671
Male	314,450	57.11	157,147	49.98	157,303	50.02	
Female	236,130	42.89	118,143	50.03	117,987	49.97	
**Age (years)**							0.961
50–59	325,450	59.11	162,716	50.00	162,734	50.00	
60–69	225,130	40.89	112,574	50.00	112,556	50.00	
**Monthly salary (NT$**^**a**^**)**							0.845
Low income	11,586	2.10	5748	49.61	5838	50.39	
≤ 17,280	32,209	5.85	16,020	49.74	16,189	50.26	
17,281-22,800	233,047	42.33	116,651	50.05	116,396	49.95	
22,801-28,800	107,277	19.48	53,665	50.02	53,612	49.98	
28,801-36,300	80,211	14.57	40,130	50.03	40,081	49.97	
≥ 36,301	86,250	15.67	43,076	49.94	43,174	50.06	
**Urbanization level of residence area**							0.891
Level 1	117,180	21.28	58,542	49.96	58,638	50.04	
Level 2	162,018	29.43	81,027	50.01	80,991	49.99	
Level 3	86,586	15.73	43,364	50.08	43,222	49.92	
Level 4	100,395	18.23	50,305	50.11	50,090	49.89	
Level 5	17,408	3.16	8679	49.86	8729	50.14	
Level 6	35,723	6.49	17,752	49.69	17,971	50.31	
Level 7	31,270	5.68	15,621	49.96	15,649	50.04	
**CCI**^**b**^							0.511
0	251,400	45.66	125,477	49.91	125,923	50.09	
1	122,117	22.18	61,131	50.06	60,986	49.94	
2	82,626	15.01	41,479	50.20	41,147	49.80	
≥ 3	94,437	17.15	47,203	49.98	47,234	50.02	

a. *NT$* New Taiwan dollar; b. *CCI* Charlson comorbidity index.

Table 4 shows the comparison of the uptake of CRC screening between people with and without disability after matching. The χ^2^ test was used to compare the observation group with the control group. After testing, the percentage of people with disability receiving CRC screening (22.47%) was lower than that of people without disability (27.18%) (*P* <  0.05). The percentage of people in each category of disability receiving CRC screening was analysed, and the results showed that the highest percentage was found in people with intractable epilepsy (39.03%), while the lowest was in people with dementia (12.24%), which is similar to the results before matching. The number of people with disability was compared with that in the general population in terms of CRC screening. Among those with low income, the percentage of people with disability receiving CRC screening (20.97%) was slightly higher than that of people without disability (19.45%), but when distinguishing the subjects by all other variables, the percentage of people with disability receiving CRC screening was lower than that of people without disability. The subgroups with CCI ≥ 3 showed the largest difference (−11.12%) in the rate of CRC screening uptake between people with disability (15.94%) and without disability (27.06%).

**Table 4:** Comparison of the Use of FIT for CRC Screening between People with and without Disability after Matching

			Without disability (*N* = 275,290)				With disability (*N* = 275,290)				
			No FIT	FIT^a^			No FIT		FIT		
	N	%	N	%	N	%	N	%	N	%	*p*-value
**Total**	550,580	100.00	200,478	72.82	74,812	27.18	213,438	77.53	61,852	22.47	< 0.001
**Sex**											
Male	314,450	57.11	117,974	75.07	39,173	24.93	123,868	78.74	33,435	21.26	< 0.001
Female	236,130	42.89	82,504	69.83	35,639	30.17	89,570	75.92	28,417	24.08	< 0.001
**Age (years)**											
50–59	325,450	59.11	105,844	65.05	56,872	34.95	113,333	69.64	49,401	30.36	< 0.001
60–69	225,130	40.89	94,634	84.06	17,940	15.94	100,105	88.94	12,451	11.06	< 0.001
**Monthly salary (NT$**^**b**^**)**											
Low income	11,586	2.10	4630	80.55	1118	19.45	4614	79.03	1224	20.97	0.045
≤ 17,280	32,209	5.85	11,984	74.81	4036	25.19	13,486	83.30	2703	16.70	< 0.001
17,281-22,800	233,047	42.33	86,836	74.44	29,815	25.56	92,579	79.54	23,817	20.46	< 0.001
22,801-28,800	107,277	19.48	39,300	73.23	14,365	26.77	41,872	78.10	11,740	21.90	< 0.001
28,801-36,300	80,211	14.57	28,509	71.04	11,621	28.96	30,176	75.29	9905	24.71	< 0.001
≥ 36,301	86,250	15.67	29,219	67.83	13,857	32.17	30,711	71.13	12,463	28.87	< 0.001
**Urbanization level of residence area**											
Level 1	117,180	21.28	43,212	73.81	15,330	26.19	45,845	78.18	12,793	21.82	< 0.001
Level 2	162,018	29.43	58,012	71.60	23,015	28.40	61,078	75.41	19,913	24.59	< 0.001
Level 3	86,586	15.73	31,725	73.16	11,639	26.84	33,883	78.39	9339	21.61	< 0.001
Level 4	100,395	18.23	37,010	73.57	13,295	26.43	39,444	78.75	10,646	21.25	< 0.001
Level 5	17,408	3.16	6304	72.64	2375	27.36	6716	76.94	2013	23.06	< 0.001
Level 6	35,723	6.49	12,884	72.58	4868	27.42	14,057	78.22	3914	21.78	< 0.001
Level 7	31,270	5.68	11,331	72.54	4290	27.46	12,415	79.33	3234	20.67	< 0.001
**CCI**^**c**^											
0	251,400	45.66	93,789	74.75	31,688	25.25	95,965	76.21	29,958	23.79	< 0.001
1	122,117	22.18	43,133	70.56	17,998	29.44	45,505	74.62	15,481	25.38	< 0.001
2	82,626	15.01	29,125	70.22	12,354	29.78	32,262	78.41	8885	21.59	< 0.001
≥ 3	94,437	17.15	34,431	72.94	12,772	27.06	39,706	84.06	7528	15.94	< 0.001
**Dental calculus cleaning**											
No	382,333	69.44	135,329	77.28	39,797	22.72	169,483	81.79	37,724	18.21	< 0.001
Yes	168,247	30.56	65,149	65.04	35,015	34.96	43,955	64.56	24,128	35.44	0.043
**Adult health examination**											
No	379,637	68.95	140,250	78.95	37,383	21.05	167,375	82.86	34,629	17.14	< 0.001
Yes	170,943	31.05	60,228	61.67	37,429	38.33	46,063	62.85	27,223	37.15	< 0.001
**Category of disability**											
Moving Functional Limitation	135,434	24.60					103,801	76.64	31,633	23.36	
Internal organ function loss and related disabilities	36,775	6.68					29,423	80.01	7352	19.99	
Hearing impairment	26,716	4.85					19,700	73.74	7016	26.26	
Multiple disabilities	21,919	3.98					18,602	84.87	3317	15.13	
Chronic mental health conditions	22,128	4.02					15,740	71.13	6388	28.87	
Visual impairment	16,430	2.98					12,981	79.01	3449	20.99	
Intellectual and developmental disability	8326	1.51					7070	84.91	1256	15.09	
Dementia	3521	0.64					3090	87.76	431	12.24	
Vocal and speech impairment	2318	0.42					1802	77.74	516	22.26	
Motion and balance impairment	770	0.14					596	77.40	174	22.60	
Facial disfigurements	426	0.08					289	67.84	137	32.16	
Intractable epilepsy	351	0.06					214	60.97	137	39.03	
Rare diseases	85	0.02					66	77.65	19	22.35	
Autism	57	0.01					42	73.68	15	26.32	
Others^d^	34	0.01					22	64.71	12	35.29	

a. *FIT* faecal immunochemical testing; b. *NT$* New Taiwan dollar; c. *CCI* Charlson comorbidity index; d. Others: chromosomal abnormalities, inborn errors of metabolism, and congenital defects.

Table 5 shows the results of conditional logistic regression analysis with the control variables on the probability of CRC screening between people with and without disability (who met the CRC screening criteria). According to Model A, in general, the OR of undergoing CRC screening in people with disability was significantly lower than that in people without disability (OR = 0.88, 95% CI = 0.87–0.89). This 12% difference in the OR was statistically significant (*P* <  0.05). In Model B, this study compared the differences in the OR of receiving CRC screening between the general population and people with different categories of disability. After controlling for different variables, the OR of receiving CRC screening in the four categories of disability (intellectual and developmental disability, dementia, multiple disabilities, moving functional limitation, and internal organ function loss and related disabilities) was significantly lower than that in the general population (OR = 0.53, 0.55, 0.62, 0.81, 0.83), while the OR in the other four categories of disability (chronic mental health conditions, visual impairment, and intractable epilepsy) was significantly higher than that in the general population (OR = 1.04, 1.09, 1.53, respectively). The probability of receiving CRC screening in people with different categories of disability was different from that of the general population, and the differences were statistically significant (*P* <  0.05).

**Table 5:** Factors Associated with the Use of FIT for CRC Screening in People with and without Disability

	Model A^a^				Model B^a^			
	OR	95% CI		*p*-value	OR	95% CI		*p*-value
**Disability**								
Without (ref)	1.00	–	–	–				
With	0.88	0.87	0.89	< 0.001				
**Category of disability**								
Without disability (ref)					1.00	–	–	–
Moving functional limitation					0.81	0.77	0.84	< 0.001
Internal organ function loss and related disabilities					0.83	0.81	0.86	< 0.001
Hearing impairment					0.94	0.83	1.06	0.279
Multiple disabilities					0.62	0.59	0.65	< 0.001
Chronic mental health conditions					1.04	1.01	1.08	0.035
Visual impairment					1.09	1.05	1.13	< 0.001
Intellectual and developmental disability					0.53	0.49	0.56	< 0.001
Dementia					0.55	0.49	0.62	< 0.001
Vocal and speech impairment					0.90	0.89	0.92	< 0.001
Motion and balance impairment					0.95	0.78	1.16	0.636
Facial disfigurements					1.17	0.92	1.50	0.208
Intractable epilepsy					1.53	1.18	1.98	0.001
Rare diseases					0.76	0.42	1.35	0.347
Autism					0.85	0.42	1.73	0.653
Other^b^					1.45	0.64	3.32	0.376

a. Both Model A and Model B controlled for seven control variables, namely, sex, age, monthly salary, urbanization level of the residential area, comorbidity severity, dental calculus cleaning, and free adult health examination; b. Others: chromosomal abnormalities, inborn errors of metabolism, and congenital defects.

## Discussion

Under Taiwan’s NHI system, among the people with disability who are eligible for free CRC screening, only 21.84% were screened. This study also found significant differences in the probability of CRC screening among people with different categories of disability who were eligible for free CRC screening. For example, the probability of receiving CRC screening in people with cognitive disability and dementia (OR = 0.72, 0.79) was significantly lower than that in people with mobility disability, while the probability of receiving CRC screening in people with mobility disability was significantly lower than that in people with intractable epilepsy, internal organ function loss and related disabilities, chronic mental health conditions, and hearing impairment (OR = 1.47, 1.34, 1.29, and 1.22, respectively), similar to the results for cervical cancer screening in Taiwan [12]. This study suggests that Taiwan should establish environments and services providing free CRC screening that are more tailored toward people with cognitive and mobility disabilities in the future.

This study found that under Taiwan’s NHI system, among people meeting the free CRC screening criteria, people with disability were less likely than people without disability to avail themselves of the free screening (OR = 0.88). This empirical study clearly demonstrates that people with disability experience health inequality, as reported by the WHO, but this health inequality can be improved by various policies [2]. The results of this study showed that in Taiwan, in contrast to the conclusion reached by the WHO, people with disability are not twice as likely to be denied access to health care as people without disability [2]. In another difference from the data in the WHO report, this study showed that Taiwan has already significantly increased the probability of receiving CRC screening among people with disability, though there is still room for improvement. In contrast to the results of this study, a meta-analysis including two studies showed that the probability of CRC screening in people with disability was not significantly lower than that in people without disability. Of the studies collected in that meta-analysis, factors such as heterogeneity and a small sample size [4] may have led to biased results. One of the analysed studies mentioned that some rural residents were not included, and the data collection time was brief, which might have led to overestimation of the study results, causing the probability of CRC screening in people with disability to be the same as that in people without disability [5]. To maximize the accuracy of the study results, following previous studies [27, 28], in addition to using nationwide data, this study adopted two steps (PSM and control variables) to better control for other relevant factors. In studies of other cancer screenings, health inequality in people with disability is common. A systematic retrospective study showed that the utilization rate of cancer screening in people with disability was significantly lower than that in people without disability [6], and some research on other cancers in Taiwan has also led to similar conclusions [10–13].

This study found that inequality existed not only between people with disability and the general population but also among people with different types of disability. Among people with disability who are eligible for free CRC screening, the probability of receiving CRC screening in people in the four categories of disability (intellectual and developmental disability, dementia, multiple disabilities, and moving functional limitation; OR = 0.53, 0.55, 0.62 and 0.81, respectively) was significantly lower than that in the general population. In contrast, the probability of receiving CRC screening in four other categories of disability (chronic mental health conditions, visual impairment, and intractable epilepsy; OR = 1.04, 1.09, and 1.53, respectively) was significantly higher than that in the general population. Inequality among different categories of disability was also found in a CRC screening study in the United States [29]. This empirical study found that health inequality in Taiwan is consistent with the WHO statement that the health status of people with disability is much lower than that of people without disability around the world [1]. The WHO report also indicated that the health inequality phenomenon can be overcome [2]. To help people with disability overcome certain obstacles [30, 31], Taiwan has introduced a variety of measures to increase the accessibility of medical care for people with disability, such as special outpatient clinics, accessible hospitals, home care, transportation subsidies, and assistive device subsidies [3, 32]. Regarding the relevant factor of residence area, the OR of receiving CRC screening in areas with low urbanization (level 3–6) (OR > 1) was significantly higher than that in areas with the highest urbanization (OR = 1). The results of this study support the fact that Taiwan has promoted many programmes to effectively eliminate geographical barriers and thereby improve the accessibility of health services, i.e., regular fixed medical care and regular mobile medical care provided by physicians, in remote areas. Since 1999, the integrated delivery system (IDS) policy has been promoted. Under Taiwan’s NHI system, major hospitals in urban areas are responsible for the delivery of medical care and preventive health care services in specific remote towns and villages [33]. A study on cervical cancer screening in Taiwan also obtained similar results [12]. In short, in accordance with the recommendations of the WHO [1], Taiwan has been working hard on legal, policy, institutional, and environmental ways to eliminate health inequalities between people with and without disability [3]. However, continuous improvements are still needed so that the health condition of Taiwanese people with disability can be further improved.

Because the data in this study were from secondary sources, information such as self-consciousness, knowledge, attitudes, and family history could not be obtained. People with better knowledge and attitudes or with a family disease history may be more likely to undergo CRC screening. Our study results showed differences in the uptake of CRC screening with FIT between people with and without disability, and these differences might result from people with different knowledge, attitudes, and family disease histories. In the future, we can conduct a survey study to examine the above-mentioned variables associated with the uptake of CRC screening with FIT in disabled people.

## Conclusions

Among people meeting the CRC screening criteria in Taiwan, people with disability were less likely to undergo CRC screening than people without disability. This health inequality in Taiwan is consistent with the WHO statement that the health status of people with disability is much lower than that of people without disability around the world, suggesting that continuous efforts should be made to eliminate health inequality between people with and without disability.

## Data Availability

Regarding the data availability, data were obtained from the National Health Insurance Research Database published by the Ministry of Health and Welfare, Taiwan. Due to legal restrictions imposed by the Taiwanese government related to the Personal Information Protection Act, the database cannot be made publicly available. All researchers can apply to use the databases to conduct their studies. Requests for data can be sent as a formal proposal to the Health and Welfare Data Science Center of the Ministry of Health and Welfare, Taiwan (http://www.mohw.gov.tw/EN/Ministry/Index.aspx). Any raw data are not allowed to be removed from the Health and Welfare Data Science Center. The restrictions prohibited the authors from making the minimal data set publicly available.

## Abbreviations

CRC: Colorectal cancer
FIT: Faecal immunochemical testing
NHI: National health insurance
NHIRD: National health insurance research database
NCCN: National comprehensive cancer network
PVS: Persistent vegetative state
CCI: Charlson comorbidity index
PSM: propensity score matching
NT$: New Taiwan dollar
OR: Odds ratio
